# Prevalence and Costs of Complementary and Alternative Medicine among Traumatic Patients in Iran: A Nationwide Population-based Study

**Published:** 2018-10

**Authors:** Mohammad Hosein KALANTAR MOTAMEDI, Abolfazl ABOUIE, Nima HAFEZI-NEJAD, Soheil SAADAT, Afarin RAHIMI-MOVAGHAR, Abbas MOTEVALIAN, Masoumeh AMIN-ESMAEILI, Vandad SHARIFI, Ahmad HAJEBI, Ali EBRAHIMI, Vafa RAHIMI-MOVAGHAR

**Affiliations:** 1. Trauma Research Center, Baqiyatallah University of Medical Sciences, Tehran, Iran; 2. Sina Trauma and Surgery Research Center, Tehran University of Medical Sciences, Tehran, Iran; 3. Iranian National Center for Addiction Studies (INCAS), Iranian Institute for Reduction of High Risk Behaviors, Tehran University of Medical Sciences, Tehran, Iran; 4. Dept. of Psychiatry, School of Medicine, Tehran University of Medical Sciences, Tehran, Iran; 5. Research Center for Addiction & Risky Behavior (ReCARB), Iran University of Medical Sciences, Tehran, Iran; 6. Dept. of Psychiatric, Iran University of Medical Sciences, Tehran, Iran

**Keywords:** Complementary/alternative medicine, Trauma, Population survey, Prevalence, Cost

## Abstract

**Background:**

This study was aimed to determine the prevalence, predictors and cost of CAM practitioner use among traumatic patients in Iran.

**Methods:**

This cross-sectional household survey of a nationally representative sample of Iranians 15 to 64 yr old was conducted in 2011, using a three-stage cluster sampling. Short Form Injury Questionnaire 7 (SFIQ7) was utilized through face-to-face interviews and data on demographics, history of injuries, mechanism, site and type of injury, type and place of the treatment were attained. Via telephone calls, service use and costs of treatment were also collected.

**Results:**

The prevalence of CAM practitioner use in injured people and victims seeking medical care was 0.7% and 4.1%, respectively in 3-month interval in 2011. There were no significant sociodemographic differences between victims who seek unconventional settings and those who seek conventional treatment. The most common injury description treated by CAM providers was as follows: fracture (type of injury), upper limb (site of injury), fall (mechanism of injury) and cast, splint, and physiotherapy (type of treatment). The average medical cost of CAM practitioner was US$14.7 while this amount in the conventional setting was US$195.5.

**Conclusion:**

Use of CAM is not very common among injured people in Iran. However, due to lack of formal training, CAM usage has possible side effects.

## Introduction

Injuries are among the leading causes of death and disability all over the world. In 2010, 11% of disability-adjusted life years (DALYs) worldwide were caused by injuries and DALYs from injuries increased over the last two decades (1). Moreover, according to a systematic analysis for the Global Burden of Disease Study (2013), global deaths from injuries have increased by 10.7%, from 4.3 million deaths in 1990 to 4.8 million in 2013 (2). Many injured people use complementary/alternative medicine (CAM) for their therapeutic purposes. For example, in folk bone-setting, bone-setters use their therapeutic methods to treat closed fractures, dislocations, disc injuries, muscles, tendons, ligaments and spine disclosures. Although traditional bone-setters dating back to thousands of years ago in Iran, this method is still administrated by traditional healers to treat many musculoskeletal disorders. (3).

CAM contains a wide range of therapies including homeopathy, naturopathy, chiropractic, massage, meditation, nutritional supplements and herbal remedies. “Many are well known, others are exotic or mysterious, and some are dangerous” (4). Despite the efficacy of modern medicine that reaches the remotest parts of the world, CAM persists and public interest to it had a significant increase in the last decades (5–12). CAM is used by significant proportions of the general population of a number of countries; previous studies have reported the prevalence of use of CAM at up to 65 percent of the general population (13, 14).

The prevalence of CAM was evaluated use by defined populations of patients, suffering from rheumatology diseases, HIV-infected/AIDS patients, cancer and pediatric patients, asthma and allergy, hepatitis C and diabetes (15–23). However, due to the paucity of sufficient information, the prevalence of CAM use in traumatic people seems to be unknown.

This study aimed to provide an overview of the utilization of CAM, specifically to determine the prevalence and cost of CAM practitioner use among traumatic patients by performing a survey at a national level in a random sample of Iran’s 15– 64 yr old residents in 2011. In addition, we sought revealing who uses CAM with regard to demographic and social characteristics.

## Materials and Methods

### Study design and Setting

A cross-sectional design was used to evaluate the prevalence, cost and predictors of CAM usage among injured people. This study was handled within the framework of Iranian Mental Health Survey (IranMHS) conducted using a population-based method in 2011 and included detailed assessment of injury (24).

### Target Population and Sampling

We included people of Iranian nationality ranging from 15 to 64 yr old. The exclusion criteria were living in institutions such as nursing houses and prisons, inability to answer the questions because of medical conditions and those who did not understand Persian language.

A three-stage cluster sampling was performed for this study. On the first stage, 1525 clusters as primary sampling units (PSUs) were randomly selected from the whole country and with a probability in proportion to size, i.e. the number of households and population of each province. Primary sampling units were selected according to the block enumerations of the national census of the Iranian Statistical Centre in 2006. In the second stage, all households living in each PSU were enumerated and six households in each cluster were selected by systematic random sampling. And finally, one of the family members of each household was chosen using Kish Grid tables (25). The sample size was computed based on the objectives of the national project. A total number of 9150 subjects were selected.

### Study variables and measurement

The Short Form Injury Questionnaire (SFIQ7) was utilized for assessment of injury. Its face and content validity were confirmed by injury specialists and its reliability was also confirmed by a pilot study (26).

Through face-to-face interviews, the questionnaires were completed. Subjects were asked about the occurrence of any kind of injuries regardless of its severity in the past 3 months. For each event, mechanism, site, type of injury, type and place of treatment were attained and coded to match the International Classification of Diseases, 10th revision 2012 (ICD10-2012) classifications. Details on specific coding and definitions have been described previously. (27)

To perform the analysis, we investigated two predominant settings of treatment: the unconventional setting (in which the injured people used unconventional therapy with or without seeking a medical doctor) and the conventional setting (the injured people sought any type of medical care but did not use unconventional therapies).

Data on demographic variables including age, gender, location, insurance (having medical insurance and complementary insurance), years of full-time education, family characteristics (head of household, marital status and number of children) and personal history (retired, unemployed and suicide history) were also collected.

After the first part of the survey (including the household interviews), the second part was performed to investigate the medical costs of injuries via telephone calls. Data on costs were collected and calculated in Iranian Rial (IRR) and are presented in the text and tables in US dollars (2011). Incidence and cost of non-fatal injuries in Iran have been previously reported (28).

### Statistical analysis

All analyses were performed using SPSS software (v. 22, (Chicago, IL, USA). We used One-way Kolmogorov-Smirnov (KS) test to explore the distribution of the study variables. Since the KS test values violated the hypothesis of normality for the included variables, we used Mann-Whitney U test for the numerical data analysis and Chi-Squared tests for qualitative data analysis along with odds ratio and 95% confidence interval. A *P*-value of less than 5% was considered statistically significant.

Costs are estimated per case in US dollars for Iran, 2011. Exchange rates were obtained from the Central Bank of Iran’s average rate for the year of the study (http://www.cbi.ir/exrates/rates_en.aspx). Average medical costs were calculated separately.

### Ethical approval

This study was approved by the Ethical Boards of Sina Trauma and Surgery Research Center, Tehran University of Medical Sciences.

## Results

Overall, 7886 subjects responded to the survey (response rate: 82.6%). One thousand, six hundred and fifty-seven subjects were reported as having an injury in the preceding three months. Of these, 291 subjects had experienced at least one injury over the three-month interval made them seek medical care.

In Table 1, we evaluated the use of CAM practitioner among participants seeking medical care based on age, gender, location, insurance and education, family characteristics and personal history. Among subjects who had a history of injury that seek medical care, 12 (4.1%) persons used CAM practitioner. The mean (±standard deviation) age of the participants seeking medical care who received and who did not receive CAM therapies was 35.1 (±14.8) and 31.6 (±11.6) yr, respectively. 5.0% of males, who received medical care for injuries during the three months, used CAM practices whereas 2.2% of females reported that they had received CAM practices for injuries. 6.9% of subjects living in rural areas and 2.3% of urban residents reported the use of CAM for injuries during the three-month interval. Among victims with no insurance, 6.3% of them reported the use of CAM, while 3.7% of the victims that had insurance used CAM practices. None of these differences were significant.

**Table 1:** Association between different characteristics and use of complementary/alternative medicine (CAM) practitioner among participants

***Variables***	***Category***	***Use of CAM n (%)***		***OR (95% CI)***	***P-value***
		***Yes***	***No***		
**Demographics**					
Age(yr)		35.1 ± 14.8^*^	31.6 ± 11.6^*^		0.426
Gender	Female	2 (2.2)	88 (97.8)	OR= 0.43 (0.09–2.02),(Baseline: Male)	0.354
	Male	10 (5.0)	191 (95.0)		
Location	Rural	8 (6.9)	108 (93.1)	OR= 3.17 (0.93–10.77),(Baseline: Urban)	0.071
	Urban	4 (2.3)	171 (97.7)		
**Insurance and Education**					
Insurance	No	3 (6.3)	45 (93.8)	OR= 1.73 (0.45–6.65),(Baseline: Yes)	0.425
	Yes	9 (3.7)	234 (96.3)		
Complementary Insurance	No	10 (4.4)	219 (95.6)	OR= 1.37 (0.29–6.42),(Baseline: Yes)	1.000
	Yes	2 (3.2)	60 (96.8)		
Full-time education, y	≤ 12	10 (4.1)	233 (95.9)	OR= 0.99 (0.21–4.65),(Baseline: > 12)	1.000
	> 12	2 (4.2)	46 (95.8)		
**Family Characteristics**					
Head of household	No	5 (3.1)	158 (96.9)	OR= 0.55 (0.17–1.77),(Baseline: Yes)	0.307
	Yes	7 (5.5)	121 (94.5)		
Married	No	4 (3.7)	104 (96.3)	OR= 0.84 (0.25–2.86),(Baseline: Yes)	1.000
	Yes	8 (4.4)	175 (95.6)		
Number of children	≤ 2	8 (3.3)	232 (96.7)	OR= 0.41 (0.12–1.40),(Baseline: > 2)	0.234
	> 2	4 (7.8)	47 (92.2)		
**Personal History**					
Retired	No	12 (4.2)	271 (95.8)	NA	1.000
	Yes	0 (0)	8 (100.0)		
Unemployed	No	12 (4.2)	271 (95.8)	NA	1.000
	Yes	0 (0)	8 (100.0)		
Suicide history	No	12 (4.2)	274 (95.8)	NA	1.000
	Yes	0 (0)	5 (100.0)		
**Total**		**12 (4.1)**	**279 (95.9)**		

OR (95%CI): odds ratio (95% confidence interval), NA: not applicable

* Mean ± Standard deviation

Table 2 shows the number of subjects seeking medical care in each category of treatment setting (conventional vs. unconventional) by type of injury, injured organ, mechanism of injury and type of treatment.

**Table 2:** Variables according to treatment setting

**Variables**	***Category***	***Treatment setting***				***Total***	
		***Conventional***		***Unconventional***			
		***n***	***%***	***n***	***%***	***n***	***%***
Type of Injury	Superficial wound	31	10.0	-	-	31	9.6
	Open wound	106	34.2	-	-	106	32.9
	Fracture	60	19.4	9	75	69	21.4
	Dislocation	24	7.7	3	25	27	8.4
	Internal organ toxicity	18	5.8	-	-	18	5.6
	Muscle and tendon injury	31	10.0	-	-	31	9.6
	Burns	26	8.4	-	-	26	8.1
	Amputation	2	0.6	-	-	2	0.6
	Unspecified	12	3.9	-	-	12	3.7
Injured organ	Head, neck & face	33	10.6	-	-	33	10.2
	Thorax	6	1.9	-	-	6	1.9
	Abdomen, spine & pelvis	25	8.1	-	-	25	7.8
	Upper limb	143	46.1	7	58.3	150	46.6
	Lower limb	79	25.5	5	41.7	84	26.1
	Multiple regions	22	7.1	-	-	22	6.8
	Unspecified	2	0.6	-	-	2	0.6
Mechanism of Injury	Transportation	52	16.8	1	8.3	53	16.5
	Fall	57	18.4	6	50.0	63	19.6
	Non-living mechanical force	129	41.6	1	8.3	130	40.4
	Living mechanical force	17	5.5	4	33.3	21	6.5
	Electricity, radiation & ambient air	5	1.6	-	-	5	1.6
	Heat & hot substances	23	7.4	-	-	23	7.1
	Toxic effect of substances	23	7.4	-	-	23	7.1
	Intentional self-harm	3	1.0	-	-	3	0.9
	Unspecified	1	0.3	-	-	1	0.3
Type of Treatment	Dressing	85	27.4	-	-	85	26.4
	Non-injectable medication	37	11.9	3	25	40	12.4
	Injectable medication	25	8.1	-	-	25	7.8
	Suture	66	21.3	-	-	66	20.5
	Cast/splint/physiotherapy	74	23.9	9	75	83	25.8
	Minor outpatient surgery	5	1.6	-	-	5	1.6
	Surgical operation	16	5.2	-	-	16	5.0
	Unspecified	2	0.6	-	-	2	0.6

For the conventional treatment setting, the most common type of injury was open wound (34.2%) followed by fracture (19.4%). The most prevalent injured organ was upper limb (46.1%) followed by lower limb (25.5%).

The most frequent mechanism of injury was non-living mechanical force (41.6%) followed by fall (18.4%). The most common type of treatment was dressing (27.4%) followed by cast, splint and physiotherapy (23.9%).

For the unconventional treatment setting, the most frequent type of injury was fracture (75%) followed by dislocation (25%). The most common injured organ was upper limb (58.3%) followed by lower limb (41.7%). The most prevalent mechanism of injury was fall (50%) followed by living mechanical force (33.3%). The most frequent type of treatment was cast, splint and physiotherapy (75%) followed by non-injectable medication (25%).

The average medical costs of injuries among victims seeking medical care in each category of treatment setting (conventional vs. unconventional) by place and type of treatment are presented in Table 3. The average medical cost of CAM practitioner was US$14.7 while this amount was US$195.5 in the conventional setting. For the conventional treatment setting, hospitalized injuries and surgical operations had the highest medical costs (average per case 330.2 and US$983.5, respectively) and primary care providers had the lowest medical cost (average per case US$16.1). The average medical cost of cast, splint and physiotherapy in conventional and unconventional treatment settings was 287.7 and US$14.7, respectively.

**Table 3:** Average medical cost of injuries that required medical care according to treatment setting

***Variables***	***Category***	***Treatment setting***	
		***Conventional***	***Unconventional***
Place of Treatment	Hospital	330.2	-
	Emergency department	186.9	-
	General clinic	42.7	-
	Private clinic	103.0	-
	Primary care provider	16.1	-
	CAM practitioner	-	14.7
Type of Treatment	Dressing	104.0	-
	Non-injectable medication	47.5	-
	Injectable medication	38.3	-
	Suture	72.8	-
	Cast/splint/physiotherapy	287.7	14.7
	Minor outpatient surgery	20.4	-
	Surgical operation	983.5	-
**Total**		**195.5**	**14.7**

## Discussion

Complementary and alternative medicine (CAM) is used frequently and increasingly. The reported prevalence of use of CAM by previous studies ranges from 5% to 74.8% and an increase of CAM usage has occurred in all countries investigated from 1990 through 2006 (29). In the USA, one in three (34%) general population have used at least one unconventional therapy in 1990. This value has risen to 42% in 1997 but has remained stable from 1997 to 2002 (5, 30, 31). Two representative population surveys of persons aged 15 or older living in South Australia were determined the prevalence and cost of alternative medicines and alternative practitioner use. The overall use of at least one non-medically prescribed alternative medicine has increased from 48.5% in 1993 to 52.1% in 2000 in an Australian population. Moreover, 23.3% of the general population had visited at least one alternative practitioner in 2000 (32, 33).

In 1990, a third of Americans used unconventional therapy has seen a CAM provider and has made an average of 19 visits to such providers during the preceding year. This probability of users visiting an alternative medicine practitioner has increased to 46.3% in 1997. Americans had made an estimated 425 million visits to CAM practitioner in 1990. Moreover, a 47.3% increase in total visits to alternative medicine providers in 1997 (629 million) has occurred (5, 30). Unfortunately, no representative study has investigated the prevalence and pattern of CAM usage in the Iranian general population. In an article on Persian language, the 12-month prevalence of CAM use was 52% general population. We found that the prevalence of CAM practitioner use in the Iranian injured people and victims seeking medical care was 0.7% and 4.1%, respectively in a 3-month interval in 2011.

In 1997, in USA, total out-of-pocket expenditures relating to alternative therapies had exceeded 1997 out-of-pocket expenditures for all US physician services (5). In addition, the expenditure on alternative therapies was nearly four times the public contribution to all pharmaceuticals in Australia in 2000 (33).

“The knowledge of CAM is mostly inherited from experiences over decades and self-practiced by their holders rather than in a written resource” (34). The reasons for popularity of CAM use most certainly are complex (35). Americans who used CAM were more likely to do so because they had believed that CAM combined with conventional medical treatments would help and/or be interesting to try (36).

In this study, there were no statistically significant sociodemographic differences between traumatic patients who seek unconventional settings and conventional treatment. The most likely users of CAM in the general population are women, middle-aged, and more educated (29). However, the use of unconventional therapy was not limited to any narrow segment of US society (30).

In our study, 25% of injured people seeking unconventional care reported that CAM practitioners used non-injectable medication for their care, whereas the majority of them (75%) reported that traditional healers used cast, splint or physiotherapy for their treatment. In Traditional Chinese Medicine, also, some topical agents have been popular for the treatment of minor injuries like sprains and avulsions (37). Folk bone-setting has been created along with Iranian traditional medicine. Although traditional bone-setting dates back to thousands of years ago in Iran, this method is still administrated by traditional healers to treat many musculoskeletal disorders (3). Despite the fact that musculoskeletal injuries are prevalent in developing countries, access to high-quality conventional treatment is not widespread. Traditional bone setters (TBS) serve to cover the gap and play a significant role in primary fracture care in developing nations, but the nature and quality of their therapy are largely understudied (38–40). The most common problems treated by them are fractures and dislocations (41). In Nigeria, about 85% of patients with fractures referred to traditional bone setters (42) but in our study, 13% of patients with fractures and 11% of patients with dislocations referred to traditional healers.

Following interviews with some Iranian bonesetters, these bonesetters had no academic education and are trained at household level orally. Their knowledge is just based on their therapeutic experiences (3).

Several studies identified the following as reasons for preference of TBS: cheaper fees (in our results: average US$14.7 vs. US$287.7 per case), easy accessibility, quick service, cultural belief, utilization of incantations and pressure from friends and families (41).

Due to lack of formal training among TBS, their practice is associated with so many problems. The major pathology leading to amputation in Nigeria was gangrene due to inappropriate splint age of fractures by traditional bone setters (43).

One of the limitations of our study was related to its population-based design; recall bias may have resulted in an underestimation of the appropriate prevalence of CAM use. Another limitation of our study was the fact that we did not perform a reliable diagnostic method to check the answers of the patients. Our study addressed the prevalence and cost of CAM practitioner use among injured people; however, further studies shall examine the complications following CAM use and appropriate training programs for CAM providers in Iran.

## Conclusion

We showed that use of CAM among Iranian traumatic victims is not very popular. However, due to lack of formal training, CAM usage has possible sequels.

## Ethical considerations

Ethical issues (Including plagiarism, informed consent, misconduct, data fabrication and/or falsification, double publication and/or submission, redundancy, etc.) have been completely observed by the authors.
